# The Head Direction Cell System and Behavior: The Effects of Lesions to the Lateral Mammillary Bodies on Spatial Memory in a Novel Landmark Task and in the Water Maze

**DOI:** 10.1037/bne0000106

**Published:** 2015-10-26

**Authors:** Bruce Harland, Emma R. Wood, Paul A. Dudchenko

**Affiliations:** 1 Psychology, School of Natural Sciences, University of Stirling, and Centre for Cognitive and Neural Systems, University of Edinburgh; 2 Centre for Cognitive and Neural Systems, University of Edinburgh; 3 Psychology, School of Natural Sciences, University of Stirling, and Centre for Cognitive and Neural Systems, University of Edinburgh

**Keywords:** spatial learning, landmarks, navigation, sense of direction, orientation

## Abstract

The head direction system is composed of neurons found in a number of connected brain areas that fire in a sharply tuned, directional way. The function of this system, however, has not been fully established. To assess this, we devised a novel spatial landmark task, comparable to the paradigms in which stimulus control has been assessed for spatially tuned neurons. The task took place in a large cylinder and required rats to dig in a specific sand cup, from among 16 alternatives, to obtain a food reward. The reinforced cup was in a fixed location relative to a salient landmark, and probe sessions confirmed that the landmark exerted stimulus control over the rats’ cup choices. To assess the contribution of the head direction cell system to this memory task, half of the animals received ibotenic acid infusions into the lateral mammillary nuclei (LMN), an essential node in the head direction network, while the other received sham lesions. No differences were observed in performance of this task between the 2 groups. Animals with LMN lesions were impaired, however, in reversal learning on a water maze task. These results suggest that the LMN, and potentially the head direction cell system, are not essential for the use of visual landmarks to guide spatial behavior.

Head direction cells are neurons in the mammalian brain whose firing is tuned to individual directions in the animal’s environment. These neurons are found in an interconnected series of brain regions, but their behavioral function is unclear. To test this, one approach has been to correlate changes in head direction cells with changes in behavior (Dudchenko & Taube, 1997; Golob, Stackman, Wong, & Taube, 2001; Muir & Taube, 2004; Valerio & Taube, 2012; van der Meer, Richmond, Braga, Wood, & Dudchenko, 2010). However, such work has yielded mixed results, with studies finding a correlation between head direction cells and spatial behavior, and others showing a lack of correlation. A second, more causative approach has been to remove or inactivate key structures in the head direction circuit, and to assess the ensuing changes in spatial behavior (e.g., Vann, 2005; Taube et al., 1992; Wilton, Baird, Muir, Honey, & Aggleton, 2001). This is the method of the current study.

The head direction cell signal is thought to arise in the connections between the dorsal tegmental nucleus and the lateral mammillary nucleus (LMN; Bassett & Taube, 2001; Sharp et al., 2001), though it requires vestibular inputs (Stackman et al., 2002), and receives inputs from other brain stem structures (Clark et al., 2009; see Winter & Taube, 2014 for review). From the LMN, the head direction (HD) signal is passed to other structures including the anterodorsal thalamic nuclei (AD; Blair et al., 1998, 1999) and, potentially, the nucleus reuniens (Jankowski et al., 2014). From the AD, the signal may pass to the postsubiculum (Goodridge & Taube, 1997), and then to the medial entorhinal cortex and the retrosplenial cortex. However, HD cells have been described in areas adjacent to these regions (e.g., the parasubiculum (Boccara et al., 2010), the anteroventral thalamus (Tsanov et al., 2011), and the lateral dorsal thalamus (Mizumori & Williams, 1993)), and it is likely that the precise pathway by which head direction information is processed is not fully understood.

Nonetheless, one approach to disrupting the head direction cell system has been to lesion or inactivate the LMN (Vann, 2005, 2011). Doing so abolishes the directional firing in the anterior dorsal thalamus (Goodridge & Taube, 1997; Blair et al., 1998, 1999), which in turn is necessary for normal head direction cell firing in the postsubiculum, and for both head direction and tuned grid cell activity in the medial entorhinal cortex (Winter, Clark, & Taube, 2015).

Lesions of the lateral mammillary nucleus yield surprisingly modest and transient spatial impairments, at least when compared with complete mammillary body or mammillothalamic tract lesions (Vann & Aggleton, 2003, 2004; for review see Vann, 2010). LMN lesions have no effect on a traditional T maze alternation task known to be sensitive to complete mammillary body lesions, and produce only a mild and transient effect on a spatial working memory task in a water maze (Vann, 2005). Subsequent work confirmed this lack of effect on a single T maze, though a modest impairment was observed when LMN-lesioned animals alternated across adjacent mazes (Vann, 2011). The latter task may be solved by the animal alternating directions (Douglas, 1966), and thus could require the head direction cell system. Vann (2011) also found that LMN-lesioned animals showed only a transient impairment in a water maze task that could be solved on the basis of its geometry. A similar transient deficit was reported after lesions of the AD, which included damage to the adjacent dorsal anteroventral nuclei in both working memory and reference memory water maze tasks (Van Groen et al., 2002).

Taken together, these studies suggest that the removal of the HD system results in a transient deficit in allocentric learning. This is surprising, given that head direction cells exhibit one of the strongest signal-to-noise ratios in the brain, and that they are present in several interconnected brain regions.

It is likely, however, that spatial tasks can be solved using different constellations of information (e.g., Dudchenko, 2001). Devising a task that specifically requires the use of heading direction would allow more precise assessment of the contribution of this system to navigation. Alternation across parallel T mazes may rely on a sense of direction (Douglas, 1966; Dudchenko & Davidson, 2002), and such alternation is sensitive to lesions of the LMN (Vann, 2011). Also, navigation in displaced water mazes may be solved using a directional sense (Hamilton et al., 2008), and performance on this task is disrupted by inactivation of the anterior dorsal thalamus in mice (Stackman, Lora, & Williams, 2012) or lesions of the dorsal tegmental nucleus in rats (Clark et al., 2013).

In the current experiment, we devised a novel landmark navigation task that capitalized on the finding that the spatial tuning of place cells, head direction cells, and grid cells can be anchored to a visual landmark in a cylindrical environment (Hafting, Fyhn, Molden, Moser, & Moser, 2005; Muller & Kubie, 1987; Taube, Muller, & Ranck, 1990). Essentially, this task involved the animal selecting a hidden reward placed in a fixed location relative to a visual landmark in a cylindrical environment. We predicted that if the head direction cell system is necessary for the association between a visual landmark and a goal direction, then damage to the LMN should impair spatial accuracy on such a task. Our results show that the visual landmark exerts stimulus control over the animal’s’ behavior in this task, but that the LMN is not necessary for its performance. The LMN does appear to be necessary, however, for flexible learning in the Morris water maze.

## Method

### Participants

Sixteen male Lister Hooded rats (Charles River Laboratories, United Kingdom) weighing 250–300 g at the start of the experiment, served as participants for this study. The rats were housed 4 to a cage and maintained in a 12-hr light/dark cycle environment. During the experiment, all rats were food restricted to ∼85% of their free feeding weight and allowed free access to water. All procedures complied with the United Kingdom Animals Scientific Procedures Act, 1986, the European Communities Council Directive of November 24, 1986 (86/609/EEC), and the American Psychological Association’s guidelines for ethical conduct in the care and use of nonhuman animals in research.

### Apparatus

#### Spatial landmark task

**Figure 1 fig1:**
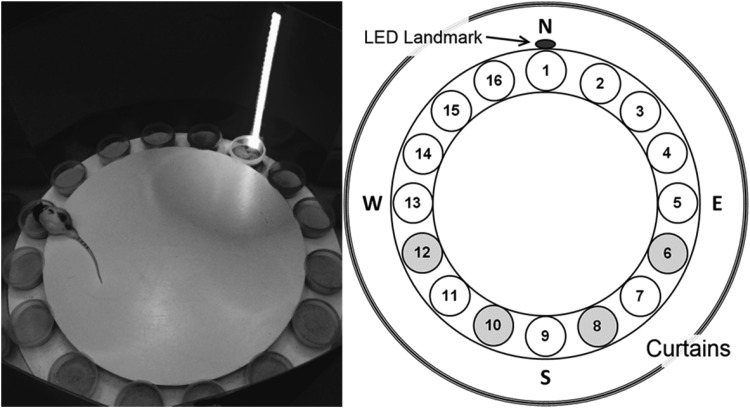
Layout of the spatial landmark task. Rats were trained to locate a single rewarded cup of sand from among 16 cups. The rewarded cup maintained the same angular association to the LED landmark throughout training, although different animals were trained on different associations (shaded cups in right plot).

Rats were tested in a cylindrical apparatus with a salient vertically oriented light-emitting diode (LED) cue that served as a landmark (see Figure 1). The cylinder floor was 1 m in diameter, made of wood, and painted white. The cylinder wall was 56.3 cm high and made from transparent Plexiglas. Black paper sheathing was attached to the outside surface of the wall to prevent visual access to potential extra maze landmarks. Two adjacent LED light strips (0.9 cm each) were attached to the cylinder wall in a vertical line within the black paper. The LED landmark was plugged into a power box allowing the light level to be adjusted. Sixteen plastic cups (11.5 cm diameter, 7 cm high) were evenly spaced every 22.5° along the perimeter of the cylinder floor, with one placed directly in front of the LED landmark. Each cup was half filled with sand that had been mixed with ground up reward (chocolate Weetos cereal loop, Weetabix, United Kingdom). A white, wooden circular central platform (76 cm diameter, 1.9 cm high) took up most of the floor of the cylinder, and it was placed on a support that raised it 3.9 cm off of the cylinder floor. This internal platform created a radial gap along the cylinder periphery into which the sand cups fit securely. The cylinder was centered within a black curtained enclosure (2.2 m diameter), with three possible entry points. These entry points were closed during experimentation. Overhead, a white sheet provided a false ceiling.[Fig-anchor fig1]

#### Water maze task

Subsequent behavioral training was conducted in a 2-m-diameter water maze located in a separate room. The water was maintained at 25 ± 1°C, made opaque by the addition of 200 ml of latex solution, and changed daily using an automatic filling and draining system. An escape platform of 11 cm diameter was submerged approximately 1 cm below the water so as to be hidden from view at the water surface. White curtains that could be pulled around the pool to occlude extra maze cues were collected together at one point north-east of the pool, serving as one of a number of extra maze cues (others included a section of caging, cupboards, doors, and 3-D objects affixed to the laboratory room walls). The animal’s swimming behavior was monitored by an overhead video camera connected to a video recorder and an online data acquisition system (Watermaze, Actimetrics, Wilmette, IL) that can digitize the path taken by the animal and then compute various spatial parameters (e.g., latency, swim speed, time in zone around platform, etc.). The data acquisition system was located in an adjacent room.

### Procedure

#### Spatial landmark task

At the start of a session, the rat was removed from its home cage, situated in the holding area outside the experimental room, and placed in an opaque black holding bucket. This was covered by a white sheet and carried into the curtained experimental enclosure and placed on a stool. The stool was moved to a new position every day. On each trial, the rat was lifted out of the bucket and placed in the center of the maze from (and also facing) one of four compass directions in relation to the LED landmark (*N, S, E, W*).

Rats were initially trained in the cylinder with eight sand cups, spaced equally on the cylinder periphery. Rats were first shaped to dig for the buried cereal loop (chocolate Weetos, Weetabix, United Kingdom) in the cup by having the loop available on the surface of the sand, and then partially burying it for several retrievals. The animals were trained until they dug for a completely buried reward for 10 trials in a daily session lasting a maximum of 10 min. Initially, each trial consisted of as many cup choices (displacement of the sand in a cup with the paws or snout) as needed until the rewarded cup was found. For each rat, one fixed cup location contained the food reward buried approximately half way through the sand. To encourage a spatial association between the rewarded cup location and the LED landmark (as opposed to a beacon strategy), we used locations that were not immediately adjacent to the landmark (see Figure 1).

Prior to each training session, the cylinder and the black outer wallpaper were rotated independently. The sand cups were switched with one another, and the LED landmark was moved to a different part of the wall (always directly behind a cup).

After at least 13 training sessions, eight additional cups of sand were added to the cylinder. After 3 additional sessions with this, the number of choices permitted on each trial was reduced to three. Rats were tested until they reached a criterion of two sessions in a row of at least 8 out of 10 rewarded trials with average errors per trial of less than 1.0 over those two sessions. Any rat unable to reach the criterion after 17 sessions was removed from the study (*n* = 2). Between trials, the rewarded cup was switched with a random other cup and rebaited.

#### Odor and landmark probe sessions

To test whether rats selected the correct sand cup based on its spatial association with the landmark, as opposed to other cues, two probe sessions were run. In one session, the odor probe, trials were run in the same way as a normal session except that on every even-numbered trial, the correct cup was not baited. For the landmark probe, 5 trials were run as usual, and then 5 additional trials were given with the LED landmark shifted by 90°. On the first of these 5 trials, neither the previously rewarded cup nor the rotationally correct cup were baited.

#### Surgery

Fourteen rats were allocated to either the bilateral LMN (*n* = 8) or the sham surgery (*n* = 6) groups. Animals were anesthetized with isoflurane (Abbott, United Kingdom) and placed in a stereotaxic frame with atraumatic ear bars (Kopf Instruments, United States). The head was adjusted in the frame to achieve flat skull coordinates. Anesthesia was maintained via an inhalation nose cone affixed to the mouth bar on the frame. Under sterile conditions, a midline incision was made, and the skull exposed. The lesions were made by injecting 0.45 μl per hemisphere of ibotenic acid (Tocris, United Kingdom; 10 mg/ml) dissolved in phosphate buffer (pH 7.4). A blunt 1-μl Hamilton syringe angled at 10° (with the tip pointing toward the rat’s tail) was aimed at the following coordinates relative to bregma: anterior-posterior – 4.5 mm, medial-lateral ± 1.0 mm, dorsal-ventral (DV) −9.2 mm (DV coordinate measured from dura). Ibotenic infusions were injected over the course of 10 min after which the needle was left in situ at each site for an additional 10 min. After completion of the ibotenic acid injections (or piercing of dura for sham animals), the skin was cleaned and sutured. The rats were given subcutaneous injections of small animal Rimadyl (Pfizer, United Kingdom; 0.08 ml/kg body weight) at the start of surgery and 2-ml glucose injections before and after surgery. All rats were given 10 days to recover with unlimited access to food and water, followed by 4 days of food restriction prior to returning to the experiment.

#### Postoperative testing in the spatial landmark task

Rats were tested for 16 sessions in the cue digging task. This was followed by another odor-control probe session, and then two landmark-control sessions (in one of these sessions the cue was moved 90° clockwise, in the other session it was moved 90° counterclockwise).

#### New spatial association training

Upon completion of the probe sessions above, the rats were trained on a new landmark-cup association. In an initial session with the new association, the reward was placed on the surface of the sand in a cup with a different spatial association to the landmark, and then partially buried on successive trials. Following this shaping session, rats were given 10 additional sessions with this new cup location.

#### Water-maze testing

Rats were habituated to swimming in the water maze with three visible platform sessions. In these, the maze was curtained off from the remainder of the room, and the submerged platform was made visible by resting an object upon it. During these habituation sessions, the platform location was counterbalanced (*NE* for half the animals, *SW* for the remainder). Each session consisted of four consecutive trials with an intertrial interval of ∼30 s, each with a different starting location (*N, S, E, W*) in a pseudorandom order. When the rat reached the platform, it sat for 30 s before being removed. All rats had an average per trial latency to reach the platform of less than 10 s by the end of the third habituation session. After the visible platform sessions, rats received seven hidden platform sessions in which the curtain was removed to expose the extra maze cues in the laboratory room. During these sessions, rats were assigned a fixed platform location (*NW* for half the animals, *SE* for the remainder) that remained constant for that rat. The sessions were run as before with each rat receiving 4 consecutive trials with the different starting locations. Any rat that did not find the platform within 120 s was guided to it by the experimenter. The day after the last of these sessions, the rats were given a 60-s probe session in the absence of a platform.

Following this, rats were given 10 reversal sessions, which were run identically to the previous hidden platform sessions except the rat was given a new fixed platform location in the quadrant of the pool opposite its initial placement (*SE* if previously *NW*; *NW* if previously *SE*). These reversal sessions were followed by a second 60-s probe session in the absence of a platform.

#### Histology

At the completion of testing, rats were given an overdose of pentobarbital solution (Euthatal, Merial Animal Health, Harlow, United Kingdom) and transcardially perfused with saline, followed by a 4% formalin solution. The brains were removed and stored in 4% formalin. A cryostat was used to cut 30-μm slices through the section of the brain containing the lateral mammillary bodies. Every second section was mounted on slides, dried, and stained with a standard Nissl stain procedure before being coverslipped. The lesion extent was measured using software to measure the area of the spared LMN, which was compared with the mean area for the control rats. Images of the sections were captured on a PC running Image Pro Plus (version 6.2; Media Cybernetics, USA) using a microscope (Leica DMRB, Germany) with a 2.5X objective and a QICAM camera (QImaging, Canada). For each image, the boundaries of the LMN were drawn manually and its area was calculated by the software.

## Results

### Presurgery Performance: Rats Readily Acquired the Spatial Landmark Task

**Figure 2 fig2:**
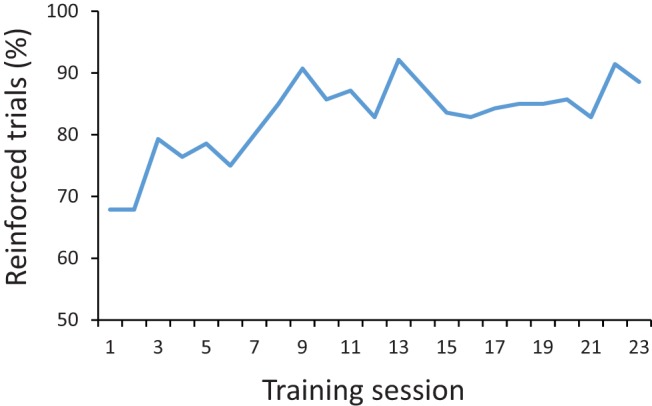
Acquisition of the spatial landmark task. Across trainings sessions, the percentage of trials in which the rats dug in the correct cup, and thereby obtained the buried reward, increased. See the online article for the color version of this figure.

After shaping, rats were given at least 23 sessions of training on the spatial landmark task. Across these sessions, the percentage of trials in which the animal obtained a reward increased significantly (Figure 2), linear trend, *F*(1, 13) = 19.2, *p* < .001. To test whether the rats were using the scent of the reward to guide their responses, we compared performance on a probe session in which half of the trials were rewarded and half were not. There was no difference in the number of errors (digs in cups other than the correct cup for each animal) in rewarded trials (*x* = 3.7 ± 1.9) compared with nonrewarded trials (*x* = 3.4 ± 1.9), *t*(13) = 0.47, *p* = .65.[Fig-anchor fig2]

**Figure 3 fig3:**
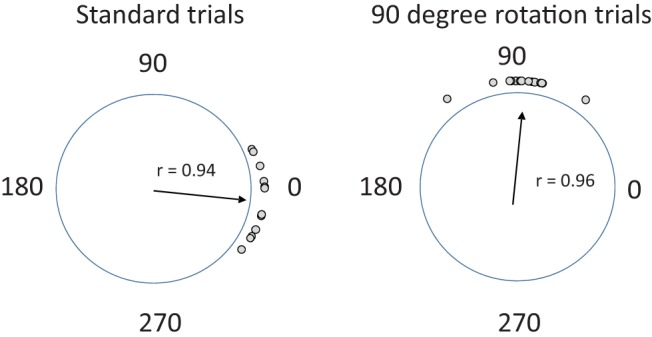
Cup choices on normal and landmark rotation trials. To calculate the average choice for each animal, cup choices for a given set of trials were converted to angles, and the mean angle of sample was calculated from these. On standard trials, choices were centered around 0°, the normalized angle of the correct cup. Following a 90° rotation of the landmark, choices were centered around 90°. This indicates that the landmark exerted stimulus control over the rats’ cup choices. Dots indicate the average for an individual animal, and the mean vector (a measure of the concentration of responses) is provided within each circle. See the online article for the color version of this figure.

A second probe session assessed the cue control exerted by the landmark. To operationalize this, the individual cup choices for each animal were converted to angles, and the mean angle of sample was calculated for the standard trials and for the trials following the 90° rotation of the landmark (Batschelet, 1981). The mean angle of sample for all choices for all animals in the standard trials was 354.7° (the correct cup being at 360°), and for the 90° rotation trials was 86.2° (the rotationally correct cup being at 90°; Figure 3). As is evident in Figure 3, the animal’s average responses were clustered around the correct and rotationally correct cups. To quantify this, we calculated the mean vector length, which varies from 0 (responses equally distributed over the 360° range) to 1 (all responses in the same direction). The mean vector lengths for all cup choices in the standard trials and in the rotation trials were *r* = .94 and *r* = .96, respectively. No difference in the number of errors (digs to incorrect cup locations) between the standard and the 90° rotation trials in the cue-control trial was found, *t*(13) = 0.54, *p* = .6.[Fig-anchor fig3]

### Histological Assessment of LMN Lesions

**Figure 4 fig4:**
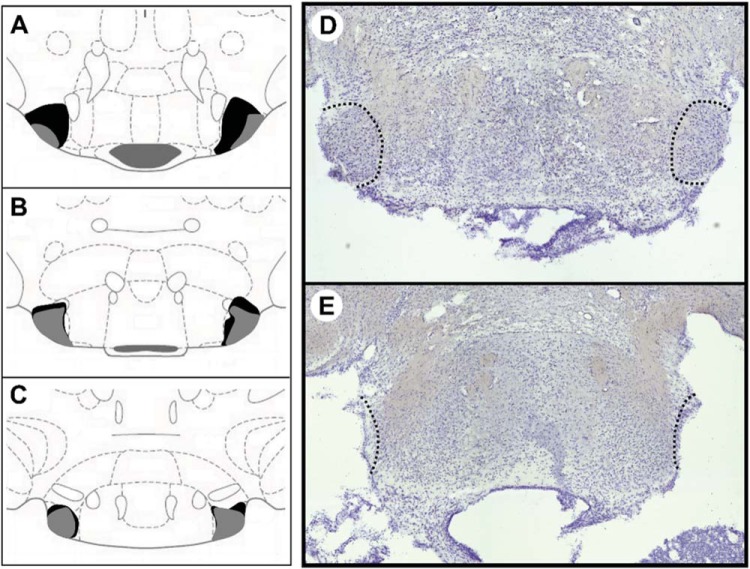
Schematic of the lateral mammillary nuclei lesions. Anterior to posterior plates showing the largest (black) and smallest (gray) lesions (A–C). Photomicrograph of a coronal section at the level of the LMN (black dashed circle; D). Corresponding photo from an animal with a LMN lesion (E). See the online article for the color version of this figure.

Bilateral infusions of ibotenic acid produced cell loss in the LMN ranging from 63.3% to 87.0% (compared with the intact LMN of the control animals; see Table 1). The mean lesion was 74.8%. The data from the animal with the smallest lesion (17.0%) and an animal with significant medial mammillary body damage were excluded from the analyses. The remaining LMN lesions included no damage to the adjacent lateral extent of the medial mammillary nuclei, although they all included moderate to severe damage to the ventral tuberomammillary nucleus, as well as marginal damage to the peduncular part of the lateral hypothalamus nucleus and the lateral part of the supramammillary nucleus (see Figure 4). The final sample sizes were Sham Lesion group, *n* = 6; LMN Lesion group, *n* = 6. Figure 4 shows the range of lesions for the anterior, middle, and posterior sections of the LMN.[Table-anchor tbl1][Fig-anchor fig4]

### Rats With Lesions of the LMN Were Able to Use a Spatial Landmark to Guide Their Behavior

**Figure 5 fig5:**
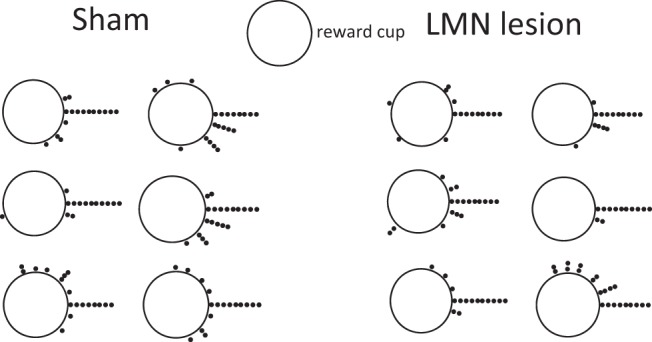
Representative postsurgery performance of animals in the sham- and LMN-lesion groups. Each circle plot represent the cup choices of an individual sham-lesioned animal on the 16th day of testing after surgery (left plots). Individual dots indicate individual cup choices. Cup choices for the animals with LMN lesions (right plots). Note that in both groups, the most frequently selected cup is the rewarded cup.

Following surgery, rats were tested for 16 sessions on the spatial landmark task. Examples of the animals’ performance are shown in Figure 5, where the cup choices for each animal on the 16th session are shown. As is evident from these plots, both the sham- and the LMN-lesioned animals show a preference for the correct cup location, and the distribution of responses looks similar across animals.[Fig-anchor fig5]

**Figure 6 fig6:**
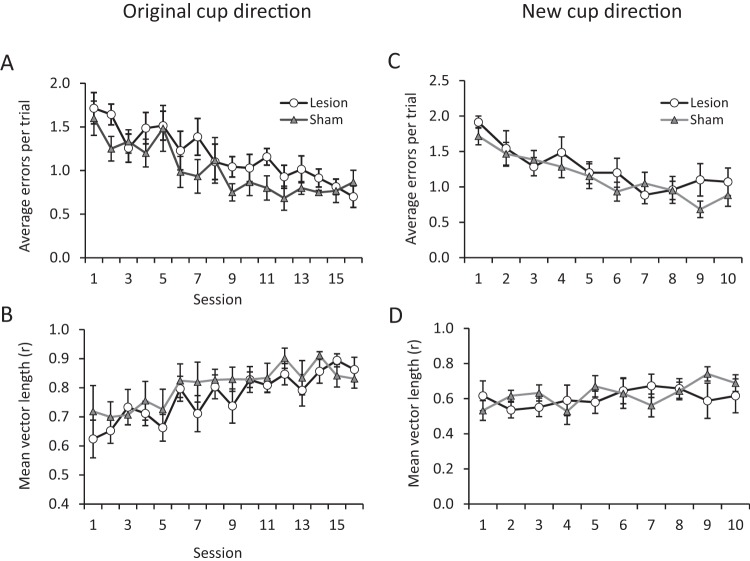
Performance on the spatial landmark task after surgery. Both lesion and sham groups decreased the number of errors made across testing sessions (mean ± standard deviation plotted in all graphs) (A). The concentration of cup choices, as measured by the mean vector, increased across testing sessions (B). When trained on a new cup location, both sham and lesioned animals performed comparably (C). The mean vector tended to increase across testing sessions with the new cup location (D).

This pattern of results was seen across the testing sessions. There were no differences between the LMN lesioned group and the sham-lesioned group in the number of errors made (Figure 6A), group effect, *F*(1, 10) = 0.76, *p* = .4; Group × Session interaction, *F*(15, 150) = 1.14, *p* = .32, or the percentage of rewarded trials, group effect, *F*(1, 10) = 0.27, *p* = .61; Group × Session interaction, *F*(15, 150) = 0.72, *p* = .76. There was also no difference in the variability of cup choices between the groups as measured by mean vector length (Figure 6B), group effect, *F*(1, 10) = 1.2, *p* = .3; Group × Session interaction, *F*(15, 150) = 0.58; *p* = .8. The two groups did not differ in the deviation between the average cup choice (as measured by the mean vector angle) and the rewarded cup position across sessions, main effect, *F*(1, 10) = 3.32, *p* = .10; Group × Session interaction, *F*(15, 150) = 1.72, *p* = .52.[Fig-anchor fig6]

The LMN-lesioned and sham animals did not differ in the percentage of trials in which the first dig was correct, *F*(1, 10) = 1.07, *p* = .33; however, an interaction between groups and sessions was observed for this measure, *F*(15, 150) = 1.93, *p* < .025. The source of this interaction was a smaller number of correct first choices by the LMN-lesioned animals relative to the controls on Days 9 and 11 (independent sample *T* tests: *p* values < .05), although performance did not differ on any other day, all *p* values > .05.

**Figure 7 fig7:**
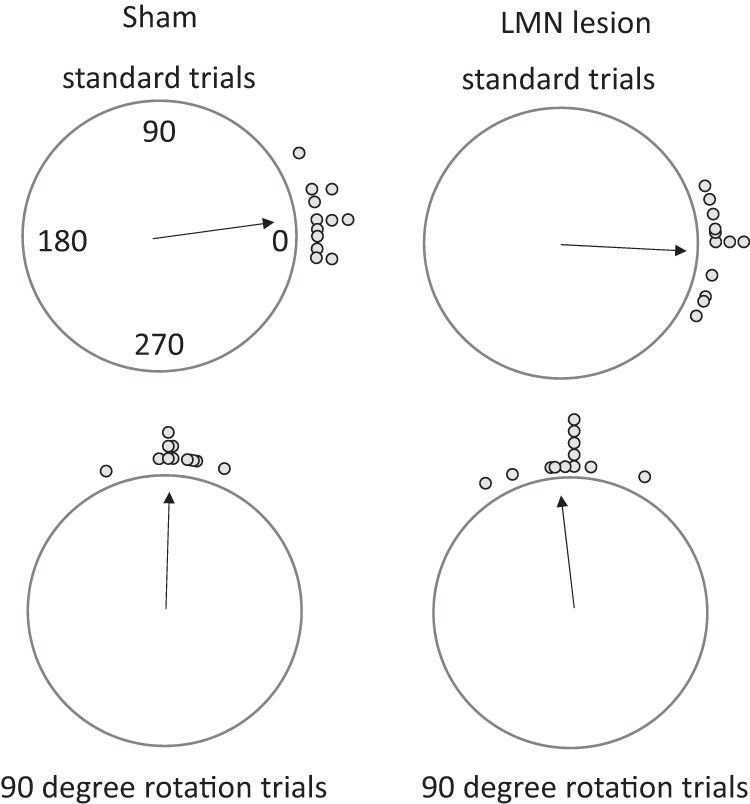
Postsurgery average direction of cup choices in normal and landmark rotation trials. Sham-lesioned animals chose the correct cup in standard trials, and tended to select a cup 90° away following the rotation of the landmark (left plots). The landmark exerted a similar control over the cup choices of animals with LMN lesions (right plots). Dots indicate the average performance of each animal in each counterclockwise rotation session and each clockwise rotation session. The latter were converted to counterclockwise directions for the sake of clarity. See the online article for the color version of this figure.

Rotation of the LED landmark within the cylinder yielded comparable rotations in cup choices for both the sham- and the LMN-lesioned animals. As shown in Figure 7, the mean cup choices for the standard trials—where the LED was in the trained position—were centered on 0° (the correct cup) for both groups (sham:5.7°; LMN lesion: 357.5°). Following the 90° rotation of the LED, the mean angle of sample for the cup choices shifted by a corresponding amount for both the sham- animals (87.1°) and the LMN-lesioned animals (93.3°).[Fig-anchor fig7]

On the reward-odor probe session, the sham-lesioned and LMN-lesioned animals did not differ in the number of errors made, *F*(1, 10) = 3.7, *p* = .09. As before surgery, there was no difference in the number of errors made when the cups were baited with reward as opposed to when they were not, *F*(1, 10) = 0.08, *p* = .79.

Rats with lesions of the LMN did not differ from sham-lesioned animals in learning a new cup location, errors, *F*(1, 10) = 0.5, *p* = .49 (see Figure 6C). In learning the new association, the groups did not differ in terms of the percentage of rewarded trials, *F*(1, 10) = 1, *p* = .34, the percentage of first dig correct trials, *F*(1, 10) = 0.21, *p* = .66, the mean vector length, *F*(1, 10) = 0.08, *p* = .78 (Figure 6D), or the mean dig vector/rewarded cup deviation, *F*(1, 10) = 0.42, *p* = .53. No correlation was observed by LMN lesion size and cumulative errors, first-time digs, rewarded trials, or deviation in either the original or new rewarded cup associations, all *p* values > 0.10.

### Rats With Lesions of the LMN Swam Slower in Acquisition of a Water Maze Task

**Figure 8 fig8:**
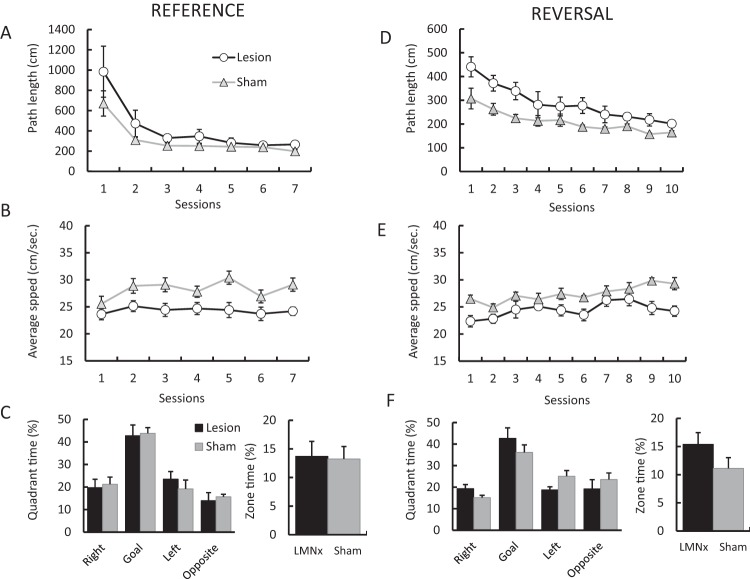
Performance on a hidden platform water maze task. Both sham- and LMN-lesioned animals decrease the path taken to the hidden platform across training sessions (mean ± standard deviation plotted in all graphs) (A). LMN-lesioned animals were significantly slower in their swimming, compared with sham-lesioned animals (B). Both groups showed a significant preference for the pool quadrant formerly containing the submerged platform in a probe session (C). Animals with LMN lesions took significantly longer paths when learning a new platform location (D). As before, the lesioned animals swam slower than the sham animals (E). Both groups showed a significant preference for the goal quadrant in a platform-removal probe session, indicating that both groups had learned the location of the hidden platform (F).

After the spatial landmark task, all rats were trained on a reference memory version of the water maze. Rats were first given 3 sessions with a visible platform, and then were tested for 7 sessions with a hidden platform. Across these 7 sessions, both sham- and LMN-lesioned animals decreased the distance traveled to find the hidden platform, session effect, *F*(6, 60) = 14.1, *p* < .001 (see Figure 8A). The two groups did not differ from each other overall, group main effect, *F*(1, 100) = 2.51, *p* = .14, nor did they differ on different sessions, Group × Session interaction, *F*(6, 60) = 0.81. The groups did not differ in latency, *F*(1, 10) = 3.63; *p* = .09. Rats with LMN lesions were significantly slower than sham-lesioned animals in terms of their average speed, *F*(1, 10) = 8.30, *p* < .02 (Figure 8B).[Fig-anchor fig8]

In the platform removal probe session, both sham- and LMN-lesioned animals showed a preference for the pool quadrant formerly containing the hidden platform (Figure 8C; quadrant effect: *F*(3, 30) = 20.2, *p* < .001). No main effect of group or interaction between the group and quadrant time was observed in the percentage of time spend in the four quadrants (main effect: *F*(1, 10) = 0.4, *p* = .55); group x quadrant interaction: *F*(3, 30) = 0.3, *p* = .85). The groups also did not differ in the amount of time spent within 20 cm of the former hidden platform location, *F*(1, 10) = .02, *p* = .89.

### Rats With Lesions of the LMN Show a Clear Impairment in Water Maze Reversal Learning

Following acquisition of the water maze, rats were given 10 training sessions with the hidden platform on the opposite side of the pool. Rats with LMN lesions took significantly longer to locate the hidden platform, *F*(1, 10) = 15.7, *p* < .0005, and this effect did not interact with training session, Group × Session interaction, *F*(9, 90) = 1.41, *p* = .20. As before, rats with LMN lesions swam slower than the sham-lesioned animals, *F*(1, 10) = 8.6, *p* < .02 (Figure 8E), and on the reversal, their path lengths were longer, *F*(1, 100) = 8.82, *p* < .02 (Figure 8D).

On the ensuing platform removal probe session, both the sham- and the LMN-lesioned animals showed a significant preference for the quadrant formerly containing the hidden platform, quadrant effect, *F*(3, 30) = 15.0, *p* < .001. As before, no differences between the groups were observed in the percentage of time in the correct quadrant, main effect, *F*(1, 30) = 1.22, *p* = .30; Group × Quadrant interaction, *F*(3, 30) = 1.54; *p* = .22. Both groups spent a similar amount of time within 20 cm of the former hidden platform location, *F*(1, 10) = 2.30, *p* = .16.

## Discussion

The aims of this study were to establish a new test for spatial landmark learning, and to test whether a key component of the head direction cell circuit, the LMN, is essential for landmark-based spatial associations. For the latter, rats with either LMN lesions or sham lesions were assessed for their memory of an association between a salient visual landmark and a hidden reward. Rats with LMN damage were able to use a spatial landmark to guide behavior with the same accuracy as control animals. However, rats with LMN lesions were impaired in reversal learning in a water maze. We consider each of these findings below.

### Performance of the Spatial Landmark Task

The behavioral task used in the current study was designed to capitalize on the findings that spatial firing of place cells, head direction cells, and grid cells has been shown to be anchored to salient visual landmark within a cylindrical environment (Hafting et al., 2005; Muller et al., 1987; Taube et al., 1990). Similar stimulus control over spatial behavior has been shown in a pellet chasing task (Lenck-Santini, Save, & Poucet, 2001). In the current study, we were interested in a task where the to-be-remembered location was in a specific direction relative to the visual landmark. Previous work in mice has shown that they will attend to multiple landmarks in a cylindrical environment depending on the task contingencies, and that these can control both behavior and place cell fields (Muzzio et al., 2009). Subsequent work with this task has shown that it requires the dorsal hippocampus (Levita & Muzzio, 2010).

In the current study, rats learned readily to dig in a cup of sand located at a fixed angle to an LED landmark. Rotation of the landmark produced a corresponding shift in the cup of sand that the animals chose. This stimulus control is consistent with that seen with rotation of landmarks in larger environments, such as the water maze or the radial arm maze (Dudchenko, Goodridge, Seiterle, & Taube, 1997; Dudchenko & Taube, 1997; Suzuki, Augerinos, & Black, 1980).

### Lesions of the LMN Have No Effect on the Spatial Landmark Task

Removal of 63%–87% of the LMN had no effect on performance of the spatial landmark task. Animals with LMN lesions were as accurate as sham-lesioned animals in their cup choices, and this spatial behavior was controlled by the salient visual landmark. Animals with LMN damage also acquired a second landmark-cup association following surgery, suggesting that plasticity in this brain region is not necessary for forming new landmark-direction associations. In both the initial task acquisition, and that of the new cup location, no correlation was observed between the animals’ accuracy and the extent of the LMN damage.

Though our prediction was that damage to the LMN, and presumably, the ensuing disruption of the head direction cell circuit, would yield deficits in orientation relative to a landmark, the lack of a deficit is consistent with previous studies. Earlier work with combined damage to the medial and lateral mammillary bodies has revealed deficits in T maze alternation (Béracochéa and Jaffard, 1995; Neave, Nagle, & Aggleton, 1997), radial arm maze working memory (Sziklas & Petrides, 2000), and water maze working memory (Vann & Aggleton, 2003). However, in these studies, the lesioned animals also showed intact performance on task variants, or improvements with training. Thus, in contrast to the effects of hippocampus lesions (e.g., Aggleton, Hunt, & Rawlins, 1986), the impairments following lesions of the mammillary nuclei are not absolute.

The effects of damage to the mammillary bodies on hippocampal place cells have been assessed by Sharp and Koester (2008). Their results do not speak directly to the current findings, as they recorded place cells in a cylinder without a polarizing landmark. Nonetheless, they did observe coherent place cells following electrolytic lesions that removed both the medial MN and LMN. Thus, some representation of location does appear to be present even in the absence of the mammillary bodies, and it may be that this is sufficient to guide some forms of spatial behavior.

It is possible, however, that the training on the spatial task prior to the LMN lesions ameliorated any potential deficit in performance, particularly a transient deficit (as seen by Vann, 2005). On this view, the LMN may normally contribute to spatial learning, but with training, spatial associations are also represented in additional brain regions. These, in turn, are sufficient for the acquisition of new spatial learning in the absence of the LMN.

### Lesions of the LMN Impair Reversal Learning on the Water Maze Task

Previous work has shown that lesions of the LMN produce an initial impairment in a version of the water maze in which a new platform location is learned each day (Vann, 2005). However, in this work, lesioned animals improved within the 4 daily trials comprising each testing session, and their performance after 8 sessions did not differ from sham-lesioned animals.

These findings may be consistent with the impairment in reversal learning observed in the current study. Though animals with LMN lesions showed significantly longer path lengths across 10 sessions of testing, they also decreased in parallel with the sham-lesioned animals. On the platform removal probe session following this training, the lesioned animals resembled the sham-lesioned animals in spending more time in the quadrant of the pool that formerly contained the hidden platform. Thus, LMN lesions in the current study and in Vann (2005) appear to reduce the animals’ capacity to form new associations between locations or directions and extra maze landmarks, at least in the water maze, but they do not abolish spatial learning.

In the only other published study in which lesions were restricted to the LMN, Vann (2011) found that such damage did not impair alternation on a single T maze, and produced a transient impairment in a water maze run in a rectangular-shaped environment. The latter effect was not seen in the initial acquisition of the water maze task in the current study, although the lesioned animals did tend to have longer latencies and path lengths than the sham-lesioned animals in the initial training sessions. The differences between these findings, however, may relate to the way in which the tasks were solved. In the Vann study, the only potential cue to the hidden platform locations was the shape of the environment, as the maze was curtained off from the rest of the laboratory. In the current study, in contrast, the shape of the environment was not a polarizing cue, and, presumably, learning of the hidden platform location reflected associations with extra maze laboratory cues (as the curtains were open). Thus, it may be that the LMN is required for the use of geometric cues, though even if these are the only cues present learning is possible (Vann, 2011).

#### Comparison between the spatial landmark and the water maze tasks

While the impairment we observed in reversal learning on the water maze suggests that lesions of the LMN impair flexible learning, such an impairment was not apparent with the learning of a new reward location on the spatial landmark test. However, these interpretations are based on different measures of performance in each maze: LMN lesion increased path length and decreased speed in the water maze, but did not affect cup choice errors or mean direction in the spatial landmark task. One possibility is that the former measures reflect an impairment in navigating toward a goal, whereas the latter reflect intact recognition of the goal location. This distinction between “getting there” and “knowing where” has been observed previously with lesions of the fornix (Whishaw, Cassel, & Jarrad, 1995). An argument in support of this view is that the LMN-lesioned animals, despite their impaired swim performance, showed an equal preference for the correct quadrant on the platform removal probe session. Thus, they were unimpaired in recognizing the correct location in the water maze task.

#### The head direction cell system and spatial learning

A puzzling aspect of the current findings and those of previous studies with lesions of the LMN (Vann, 2005, 2011) is that the impairments observed are milder than those following lesions of the upstream head direction region, the dorsal tegmental nuclei (DTN; Clark et al., 2013; Dwyer et al., 2013; Frohardt, Bassett, & Taube, 2006). There are at least two possible explanations. First, it might be that the DTN has additional functionality that renders it particularly essential for navigation. Though the DTN is a major input to the LMN (Liu, Chang, & Wickern, 1984), it contains a larger percentage of cells encoding angular head velocity (∼75% according to Bassett & Taube, 2001) compared with the LMN (∼44% according to Stackman & Taube, 1998). It may be that such differences are important in the ultimate representation of orientation.

A second possibility is that the extra maze landmarks in the current study, particularly the spatial landmark task, were more salient than the tasks used in previous studies with DTN lesions. In Frohardt et al. (2006) (nonblindfolded condition), Clark et al. (2013), and Dwyer et al. (2013) (table-top task), the landmark or landmarks that presumably guided navigation were outside the respective maze environments used. In the current study, our LED landmark was affixed to the outside wall of the environment. Thus, it might be possible that association between the correct cup location and the LED landmark is somewhat easier to make than an association between a home location or hidden platform and distal landmarks.

### Summary

The current study makes two contributions. First, we have established a novel task that is based on the use of a visual landmark to guide spatial behavior. Such a task is, perhaps, a behavioral analogue to the traditional paradigms for assessing stimulus control over the firing of spatially tuned neurons. Second, we show that the LMN are not necessary for acquisition of a landmark-direction association, though they do appear to be involved in flexible learning in the water maze. It remains possible that the LMN (and the head direction cell system) underlie navigation in the absence of landmarks (Valerio & Taube, 2012; van der Meer et al., 2010), and there is evidence that lesions of the LMN impair homing (van der Meer, 2007). However, the current results indicate that LMN are not necessary for spatial landmark learning.

## Figures and Tables

**Table 1 tbl1:** Percentage of Cell Loss in the LMN

Rat	Percentage cell loss
1	63.3
2	67.1
3	74.5
4	77.3
5	79.7
6	87.0
